# Rheological and Viscoelastic Analysis of Hybrid Formulations for Topical Application

**DOI:** 10.3390/pharmaceutics15102392

**Published:** 2023-09-27

**Authors:** Maria Natalia Calienni, Luis Manuel Martínez, Maria Cecilia Izquierdo, Silvia del Valle Alonso, Jorge Montanari

**Affiliations:** 1 Laboratorio de Bio-Nanotecnología, Departamento de Ciencia y Tecnología, Universidad Nacional de Quilmes, Bernal 1876, Argentinajorge.montanari@unahur.edu.ar (J.M.); 2 Grupo de Biología Estructural y Biotecnología (GBEyB), IMBICE (CONICET CCT-La Plata), La Plata 1906, Argentina; 3 Laboratorio de Nanosistemas de Aplicación Biotecnológica (LANSAB), Universidad Nacional de Hurlingham, Villa Tesei 1688, Argentina; 4 Comisión de Investigaciones Científicas de la Provincia de Buenos Aires (CIC), La Plata 1900, Argentina

**Keywords:** skin, topical products, liposomes, cream, gel, thermal water, rheology, viscoelasticity

## Abstract

The rheological and viscoelastic properties of hybrid formulations composed of vehicles designed for cutaneous topical application and loaded with ultradeformable liposomes (UDL) were assessed. UDL were selected for their established ability to transport both lipophilic and hydrophilic compounds through the skin, and are applicable in pharmaceuticals and cosmetics. Formulations underwent flow analysis and were fitted to the Herschel–Bulkley model due to their prevalent non-Newtonian behavior in most cases. Linear viscoelastic regions (LVR) were identified, and G′ and G″ moduli were determined via frequency sweep steps, considering the impact of temperature and aging. The formulations exhibited non-Newtonian behavior with pseudoplastic traits in most cases, with UDL incorporation inducing rheological changes. LVR and frequency sweep tests indicated predominantly elastic solid behavior, with G′ higher than G″, at different temperatures and post-production times. Tan δ values also illustrated a predominant solid-like behavior over liquid. This study provides pivotal insights into the rheological and viscoelastic features of topical formulations, emphasizing the crucial role of meticulous vehicle and formulation selection when incorporating UDL or analogous liposomal drug delivery systems.

## 1. Introduction

Ultradeformable liposomes (UDL), also known as transfersomes, emerged in the 1990s as nanodelivery systems specifically tailored for topical administration [1]. UDL exhibit unique elastic characteristics that enable them to deform and traverse the intercorneocyte pathway, enabling their penetration into the viable epidermis beyond the *stratum corneum* (SC) barrier. UDL can be obtained using well-established methods, and they possess the capability to encapsulate a diverse range of active agents within their lipidic bilayers and aqueous cores. As a versatile carrier, UDL offer the potential to deliver active compounds not only for therapeutic applications but also for cosmeceutical purposes [2].

While several studies have focused on incorporating active compounds into UDL and exploring their interactions with the skin in the form of aqueous suspensions [3,4,5,6,7,8], fewer investigations have delved into the behavior of UDL when incorporated into commonly used vehicles within the pharmaceutical and cosmetic industries [9,10]. Recognizing that formulations such as gels and creams are pivotal for creating market-ready products suitable for user self-application, a comprehensive exploration of the properties and performance of formulations is imperative. Our prior studies have demonstrated that UDL, after integration into various vehicles commonly used in the cosmetic industry, retain their ability to facilitate the penetration of loaded molecules into the viable epidermis and dermis (VED) of human skin [10]. However, the characterization of such formulations predominantly focused on the nanocarrier and the loaded substances, with less emphasis on the overall end products. A more holistic investigation should encompass the impact of UDL suspension incorporation on the vehicle matrices.

Pharmaceutical and cosmeceutical products for cutaneous application often adopt semi-solid forms such as creams, ointments, lotions, emulsions, or suspensions comprising distinct components for topical use [11,12]. Their formulation demands specific flow properties to facilitate packaging while maintaining stability over time, enabling dispensing, and ensuring appropriate application onto the skin. Consequently, rheological properties play a crucial role, directly influencing the efficacy, performance, user adherence, and overall cost-effectiveness of the therapeutic or cosmeceutical formulation [13]. Ultimately, patient acceptance, adherence to prescribed treatments, and overall healthcare expenses are influenced by intrinsic and extrinsic factors that impact the flow properties of pharmaceutical and cosmeceutical materials throughout their production, administration, and usage phases. Many consumer products and industrial formulations, including pharmaceutical and cosmeceutical products, consist of colloidal suspensions in which the suspending medium itself is a complex fluid [14]. Likewise, over recent decades, there has been a growing realization that many multi-phase compositions such as emulsions and dispersions do not adhere to the Newtonian assumption of a linear relationship between shear stress and shear rate [15]. The intricate internal structure of complex fluids can lead to nonlinear rheological responses when exposed to various processing conditions such as extrusion, coating, spraying, or lubrication [16], among others. Non-proportionality between shear rate and shear stress is a characteristic feature of non-Newtonian behavior [17]. One type of non-Newtonian fluid is pseudoplastic, a fluid whose apparent viscosity gradually decreases with an increase in shear rate, a phenomenon known as shear thinning [17]. Particularly, in a topical formulation, pseudoplastic behavior is expected, as it ensures a uniform distribution on the skin [18]. Consequently, gaining a profound understanding of the rheological behavior of these complex fluids is imperative.

Recently, we conducted a research study into the incorporation of UDL into various widely utilized vehicles within the cosmetic industry, culminating in the creation of final products containing diverse active ingredients and excipients [10]. These vehicles include creams, gels, and thermal water. This comprehensive study encompassed several biophysical aspects, including the determination of size, Z-potential, and penetration behavior on human skin. Notably, the incorporation of UDL into the studied vehicles exhibited an impact on the penetration profile of these liposomes in human skin overall, resulting in their successful traversal to the VED layers. This newfound understanding holds significant value for the formulation of versatile topical products that incorporate these nanosystems, potentially conferring unique attributes by enhancing diffusion across the SC and even within the VED. In this manner, the transported active compounds could effectively enact their effects.

Building upon the aforementioned findings and considering future implications, our study aims to delve into the rheological and viscoelastic properties of various vehicles commonly employed in the cosmetic industry. These vehicles also find application in pharmaceutical formulations, both as standalone entities and as components of commercially available products. This inquiry encompasses an exploration of how the addition of UDL suspensions influences these properties, while also accounting for variations in temperature and stability over time. We seek to unravel the intricate relationship between UDL integration and the physical behavior of the formulations, shedding light on how temperature and aging could impact the overall stability and effectiveness of the resultant products.

## 2. Materials and Methods

### 2.1. Materials

Soybean phosphatidylcholine (SPC) and sodium cholate (NaChol) were purchased from Avanti Polar Lipids (Alabaster, AL, USA) and Sigma-Aldrich (Buenos Aires, Argentina), respectively. All vehicles were donated by Luvré Pro (Buenos Aires, Argentina). All other reagents used were of analytical grade and were purchased from Anedra (Argentina). The composition of vehicles is presented in Table 1.

### 2.2. UDL Obtention and Incorporation into Topical Vehicles

UDL were prepared following previously established protocols [19]. In brief, an organic solution of SPC and NaChol (6:1 *w*/*w*) in chloroform and methanol (1:1 *v*/*v*) was evaporated into a round-bottom flask using rotary evaporation under vacuum at 40 °C. The resulting dry lipid film was then hydrated with 10 mM Tris–HCl buffer (containing 0.9% NaCl, pH 7.4) until a final concentration of 40 mg/mL of SPC was achieved. The obtained liposomes were subsequently reduced in size and lamellarity using an automated extruder (Transferra Nanosciences Inc., Burnaby, BC, Canada) with five extrusion cycles via a polycarbonate filter of 100 nm pore size under a N_2_ flux.

Various topical vehicles were employed, specifically base gel with essence (BGE), base cream (BC), a final commercial formulation cream-based (CC), and thermal water (TW). These selections were made to represent the different types of vehicles commonly used for topical application in the cosmetic industry and were based on a previous study we conducted [10]. UDL were integrated into vehicles by gently and convectively mixing with a spatula for 1 min. The chosen UDL/vehicle ratio was 100 µL of UDL for every 500 mg of BGE, BC, and CC obtaining BGE-UDL, BC-UDL, and CC-UDL, respectively, based on previous data obtained [10]. As for TW, the ratio used was 40 µL of UDL for each 60 µL of vehicle to obtain TW-UDL.

### 2.3. Rheological and Viscoelastic Assays

Vehicles both with and without UDL were compared in the studies. Samples were analyzed using a controlled-stress rheometer (AR-G2, TA Instruments, TA Instruments Ltd., New Castle, UK) with a temperature control system that involved a recirculating bath ACW 100 (JULABO GmbH, Seelbach, Germany) connected to the Peltier plate, following the protocols reported by Bucci et al. 2018 [9]. The cone-and-plate geometry had a diameter of 40 mm with a cone angle of 2° and a truncation of 55 µm. Data recording was conducted using TA Advantage Software Instruments v5.5.24. Three measurements were taken for each sample and the average value was calculated (*n* = 3). Data were analyzed using OriginPro 2019b software.

#### 2.3.1. Flow Analysis

A total of 0.7 mL of the sample was dispensed onto the plate at 20 °C and 37 °C for 2 min to allow the sample to reach the desired temperature. Subsequently, a pre-shear of 0.1 Pa was applied for 10 s, and a 30 s stabilization period followed before initiating the test. The shear rate ranged from 0.1 to 300 s^−1^. We conducted 30 experimental measurements on all the tested samples. Each shear rate was applied to the sample for 5 s, and the measurement was taken during the final 0.5 s.

#### 2.3.2. Viscoelastic Analysis

Each sample was prepared and analyzed following the flow analysis procedure described earlier. The linear viscoelastic region (LVR) was determined using a stress sweep step. The variation of the storage module (G′) was assessed by measuring the shear stress variation from 1 to 1000 Pa for both the vehicle matrices and the UDL-loaded formulations at 20 °C and 37 °C, as well as at 1 day post-production (dpp) and 30 dpp. Subsequently, a constant shear stress value of τ = 5 Pa from the LVR assay was maintained to determine the storage or elastic (G′) and loss or viscous (G″) modules using a frequency sweep step, varying the frequency from 0.1 to 257 rad/s.

### 2.4. Statistical Analysis

The data were analyzed after confirming normal distribution via the Shapiro–Wilk test. Homoscedasticity was also verified using the F-test to compare variances. One-way ANOVA and Dunnett’s multiple comparisons test were conducted to determine significant differences between UDL-loaded formulations and the vehicles (*p* < 0.05). The statistical analyses were performed using GraphPad Prism 8.0.1 software (GraphPad Software Inc., San Diego, CA, USA).

## 3. Results

### 3.1. Flow Analysis

**Figure 1:**
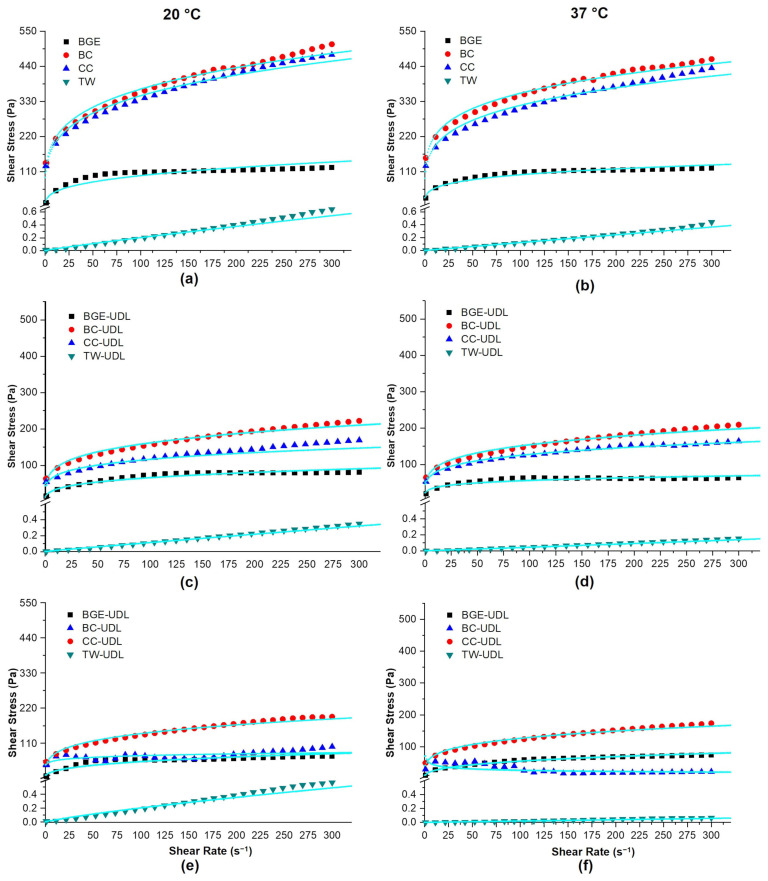
The flow behavior of vehicles at (**a**) 20 °C and (**b**) 37 °C, UDL-loaded formulations at 1 day post-production (dpp) at (**c**) 20 °C and (**d**) 37 °C, and UDL-loaded formulations at 30 dpp at (**e**) 20 °C and (**f**) 37 °C. The experimental datasets were fitted with the Herschel–Bulkley model.

In this study, the rheological properties of vehicle matrices (BGE, BC, CC, and TW) and vehicles loaded with UDL (BGE-UDL, BC-UDL, CC-UDL, and TW-UDL) at both 20 °C and 37 °C, under various shear rates, and at 1 and 30 dpp were analyzed. The flow shear stress of each formulation is shown in Figure 1.

Based on the data obtained from Figure 1, a non-Newtonian flow behavior model with pseudoplastic characteristics was adopted [20], following the Herschel–Bulkley model (Equation (1)):

τ = τ_0_ + K γ^n^ (1)

where τ is the shear stress [Pa], τ_0_ is the initial shear stress [Pa], K is the consistency index [Pa × s^n^], γ is the shear rate [s^−1^], and “*n*” is the fluidity index. The parameters of the proposed model for the different vehicles studied are provided in Table 2, while the parameters for the different UDL-loaded formulations at both temperatures are given in Table 3 (1 dpp) and Table 4 (30 dpp).

The non-proportionality between shear rate and shear stress is characteristic of non-Newtonian behavior, and pseudoplastic fluids are fluids whose viscosity decreases with an increase in shear rate (shear thinning) [17]. As expected for topical products, a pseudoplastic behavior was observed, which ensures uniform distribution on the skin [18]. The non-Newtonian behavior of the studied vehicles is also evident in the flow index “*n*” in Table 2, as all samples have a value less than one. The pseudoplastic behavior becomes more pronounced with increasing crosslinking and interaction between molecules in the different vehicles. This effect enhances the resistance of the fluids to deformation, as indicated by the consistency index (K) shown in Table 2.

In Figure 1, experimental data points and the fitting model proposed for the vehicles and formulations are presented. Figure 1a,b, which represent the vehicles without UDL, exhibited a non-Newtonian behavior with pseudoplastic characteristics for BGE, BC, and CC. The proposed model was adjusted according to the parameters from Table 2 for both tested temperatures (20 °C and 37 °C). For the TW vehicle, although its behavior was Newtonian, we employed the Herschel–Bulkley model for non-Newtonian fluids. Remarkably, we obtained “*n*” values of 0.906 at 20 °C and 0.992 at 37 °C, which are very close to 1, and the R^2^ value was greater than 0.93. The starting tensions (τ_0_) were nearly negligible. The values applied to fit Equation (1) resulted in a line that closely aligned with the experimental points for the TW vehicle. The BGE and BC vehicles exhibited higher consistency values at 37 °C than at 20 °C, whereas for CC and TW, the consistency was greater at 20 °C than at 37 °C. In a general analysis of the vehicles’ consistency, it can be observed that BC > CC > BGE >> TW for both temperatures, indicating that the structures offer greater resistance to flow as the value of the consistency (K) increases. The consistency values between BGE, BC, and CC ranged from 21.17 Pa × s^n^ to 121.05 Pa × s^n^ at 20 °C and from 32.65 Pa × s^n^ to 135.37 Pa × s^n^ at 37 °C. For TW, the consistency value can be considered the value of the dynamic viscosity of the system, adopting the values 3.1 × 10^−3^ Pa × s^n^ for 20 °C and 2.1 × 10^−4^ Pa × s^n^ for 37 °C. When comparing the dynamic viscosity values of BGE, BC, and CC with TW for both temperatures, it becomes evident that the ability to flow of the TW is much higher than the creams or gels, as expected.

Figure 1c,d depict the formulations BGE-UDL, BC-UDL, and CC-UDL at 1 dpp, showing non-Newtonian behavior with pseudoplastic characteristics, similar to BC, BGE, and CC vehicles. The proposed model was adjusted according to the parameters in Table 3 for both temperatures (20 °C and 37 °C). For the TW-UDL formulation, similar to the TW vehicle, it exhibits Newtonian behavior. Considering the criteria applied to the TW vehicle, the “*n*” values were very close to 1 (0.963 at 20 °C and 1.040 at 37 °C), with R^2^ greater than 0.93, and the starting tensions (τ_0_) were practically negligible. The values applied to Equation (1) would form a line, representing an adjustment to the experimental points obtained for the TW-UDL formulation. All formulations at 1 dpp (BGE-UDL, BC-UDL, CC-UDL, and TW-UDL) exhibited higher consistency values at 37 °C compared to 20 °C. In a general analysis of the consistency in the formulations at 1 dpp, it is evident that BC-UDL > CC-UDL > BGE-UDL >> TW-UDL for both temperatures, indicating that these structures offer greater resistance to flow as the value of consistency increases. The consistency values between BGE-UDL, BC-UDL, and CC-UDL ranged from 17.31 Pa × s^n^ to 53.85 Pa × s^n^ at 20 °C and from 23.93 Pa × s^n^ to 54.45 Pa × s^n^ at 37 °C. Regarding TW-UDL, the dynamic viscosity compared to that of the vehicle without UDL is significantly lower, with values of 1.3 × 10^−3^ Pa × s^n^ at 20 °C and 3.3 × 10^−4^ Pa × s^n^ at 37 °C. The ability to flow the TW was much higher than the creams or gels used as the base support system for formulations with liposomes.

Figure 1e,f demonstrate that BGE-UDL and CC-UDL at 30 dpp exhibited non-Newtonian behavior and pseudoplastic characteristics, similar to what was observed at 1 dpp, and also in line with the behavior of BGE and CC vehicles. These formulations showed specific adjustments to the proposed model, as indicated in Table 4 for both temperatures (20 °C and 37 °C). BC-UDL at 30 dpp did not fit the Herschel–Bulkley model. For the TW-UDL formulation, once again, it exhibited Newtonian behavior. Considering the criteria applied to the TW vehicle and to the TW-UDL at 1 dpp, the “*n*” values were 0.891 at 20 °C and 1.004 at 37 °C, both very close to 1 and with R^2^ greater than 0.89, and the starting tensions (τ_0_) were practically negligible. The values applied to the equation formed a line that closely aligned with the experimental points obtained for the TW-UDL formulation. BC-UDL and TW-UDL exhibited lower and higher consistency values at 20 °C compared to 37 °C, respectively. However, the BC-UDL formulation at 30 dpp showed alterations in its appearance, including phase separation and a color change. This phase separation could lead to wall slip, consequently causing non-uniform flow near the walls of the instrument and resulting in inaccurate measurements at certain shear rates. This might explain why the rheological behavior of BC-UDL at 30 dpp can no longer be accurately fitted to the Herschel–Bulkley model, as evident in Table 4 and Figure 1e,f. On the other hand, BGE-UDL and CC-UDL at 30 dpp exhibited similar consistency values at 20 °C and 37 °C. The consistency in the formulations at 30 dpp followed the sequence CC-UDL > BGE-UDL >> TW-UDL, for both temperatures. These results indicate that the formulation structures offer greater resistance to flow as the value of consistency increases. The consistency values between BGE-UDL and CC-UDL ranged from 10.85 Pa × s^n^ to 43.45 Pa × s^n^ at 20 °C and from 11.89 Pa × s^n^ to 42.16 Pa × s^n^ at 37 °C. In the case of TW-UDL, considering the analysis carried out for the vehicle and formulation at 1 dpp, the dynamic viscosity adopted values of 5.4 × 10^−3^ Pa × s^n^ for 20 °C and 1.8 × 10^−4^ Pa × s^n^ for 37 °C. TW and both TW-UDL at 1 and 30 dpp exhibited a far superior flow ability compared to creams or gels, as expected.

**Figure 2:**
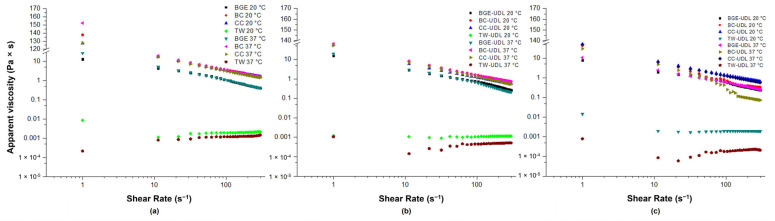
The variation of apparent viscosity with shear rate at 20 °C and 37 °C for vehicles (**a**), UDL-loaded formulations at 1 dpp (**b**), and UDL-loaded formulations at 30 dpp (**c**).

On the other hand, the viscosity behavior of vehicles and UDL-loaded formulations at 1 and 30 dpp was assessed at both 20 °C and 37 °C, as shown in Figure 2. At low shear rates, below 10 s^−1^, the viscosity of the vehicles (BGE, BC, and CC) was higher than that of the vehicles containing UDL for both temperatures. The addition of UDL to the vehicles resulted in the stabilization of the formulations at lower shear rates compared to the vehicles without UDL. The values of apparent viscosity versus shear rate clearly indicate a shear-thinning system, which is a desirable characteristic for a good topical vehicle and formulation [21,22]. On the contrary, TW and TW-UDL exhibit a practically constant viscosity value across all shear rate ranges, indicating Newtonian behavior.

### 3.2. Viscoelastic Analysis

**Figure 3:**
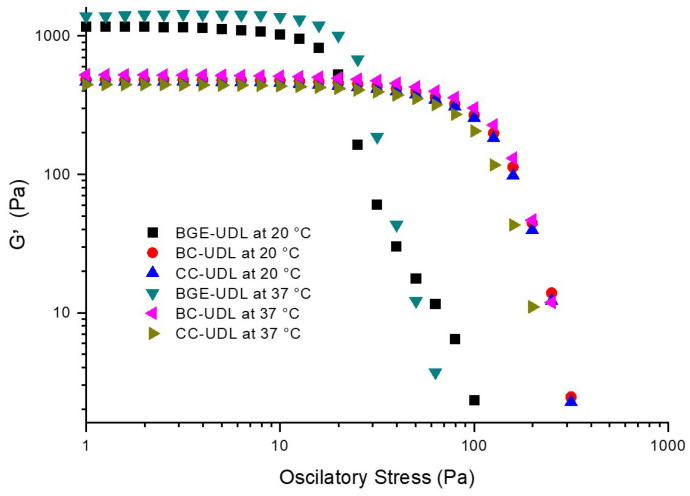
The linear viscoelastic region (LVR) for the UDL-loaded formulations at 20 °C and 37 °C at 1 dpp. Tests were performed at an angular frequency of 1 Hz.

To gain insight into the viscous and elastic behavior of our systems and the network structure formed by particle–particle interactions, we conducted oscillation sweep tests [23]. The amplitude sweep test involves two steps. Firstly, we determine the linear viscoelastic region (LVR) of the samples, which is achieved via a stress sweep at a constant frequency. The LVR represents a stress range over which G* (complex modulus) and the structure of the material remain intact. In our study, the stress sweep step was performed with a stress value (τ) of 1 Pa, which was chosen based on previous research [9] and allowed us to identify the linear viscoelastic region. El Kechai et al. (2015) also studied the viscoelastic aggregation properties of liposomes on hyaluronic acid hydrogels at 37 °C under a stress value of 10 Pa [24], which belonged to the linear viscoelastic regime where the samples did not undergo irreversible structural modifications. In our work, all tested formulations exhibited their own linear viscoelastic regions within the low stress range (τ < 5 Pa). The stress sweep step for the formulations, as shown in Figure 3, clearly revealed the linear viscoelastic region.

In Figure 3, it can be observed that the structures of BGE-UDL have a higher value of G′ (storage modulus) compared to CC-UDL and BC-UDL under the oscillatory stress range in the study (~1126 Pa > ~472 Pa and ~462 Pa, respectively) at 20 °C, and the formulations were not significantly influenced by the temperature, maintaining similar values (~1138 Pa > ~521 Pa and ~472 Pa, respectively) at 37 °C. However, BGE-UDL lost its solid-like elastic characteristic at an oscillatory stress value of ~9 Pa, while this value for CC-UDL and BC-UDL was ~60 Pa. For these formulations, as the shear stress values increase, the elastic modulus (G′) decreases, indicating changes in the material structure. In further experiments, we will explore stress (τ) values near the end of the linear viscoelastic range (LVR). Based on the data, the value selected for the frequency sweep step assay was 5 Pa.

**Figure 4:**
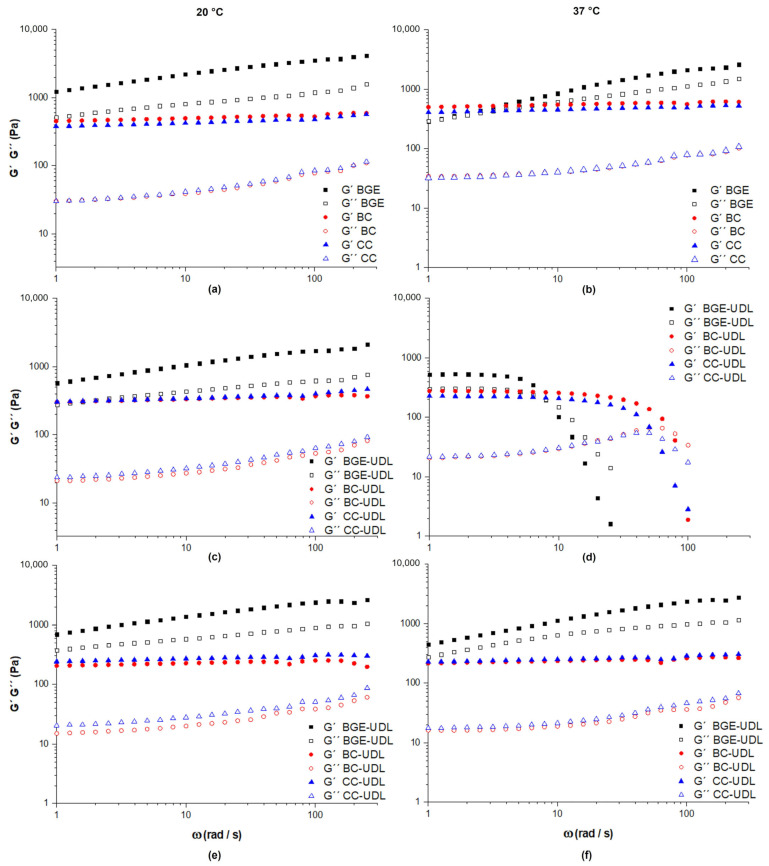
The viscoelastic curves of G′ and G″ obtained from the frequency sweep step at τ = 5 Pa for vehicles at 20 °C (**a**) and 37 °C (**b**), UDL-loaded formulations at 1 dpp at 20 °C (**c**) and 37 °C (**d**), and UDL-loaded formulations at 30 dpp at 20 °C (**e**) and 37 °C (**f**).

The frequency sweep step is another important test that provides information about the storage or elastic modulus (G′), the loss or viscous modulus (G″), and the loss tangent (tan δ). The G′ represents the energy stored and recovered in each deformation cycle, indicating the elastic behavior of the system. On the other hand, the G″ determines the energy loss of each cycle, representing the liquid-like behavior of the system. The G′ and G″ of the vehicles and UDL-loaded formulations at 1 and 30 dpp were determined via frequency sweep step assays at 20 °C and 37 °C (Figure 4).

The G′ and G″ values for BGE at 20 °C were higher than those of CC and BC, and values increased throughout the test (Figure 4a). The viscoelastic behavior of G′ and G″ for BC and CC were similar. At the frequencies studied and 20 °C, G′ was always greater than G″. The vehicles maintained their structure throughout the test, predominantly showing an elastic solid behavior over a viscous liquid one.

The temperature increment did not produce significant changes in the viscoelastic behavior of both the creams (CC and BC) and the gel (BGE) as can be seen in Figure 4b. At both temperatures, all vehicles studied maintained their elastic solid behavior throughout the test, but the values of G′ and G″ were lower at 37 °C compared to those at 20 °C for all the frequencies covered for BGE. Additionally, the gel (BGE) exhibited higher values of G′ and G″ than the creams (BC and CC) at both temperatures. Only at low frequencies and 37 °C did the G′ and G″ values of BGE become closer to those of the creams.

On the other hand, at 1 dpp and 20 °C (Figure 4c), BGE-UDL continued to display a higher elastic solid behavior compared to the CC-UDL and BC-UDL formulations, consistent with the observations made for the vehicles. Both BC-UDL and CC-UDL formulations exhibited similar viscoelastic behavior throughout the frequency sweep test. However, the incorporation of UDL at 20 °C led to changes in the G′ and G″. The values of G′ and G″ for the UDL-loaded formulations were lower than those of the vehicles throughout the test. The G′ values were higher than G″ values, indicating that the formulations maintained their structure and exhibited an elastic solid character over a viscous liquid.

When the temperature increased to 37 °C (Figure 4d), an unusual rheological response was observed for all the UDL-loaded formulations at 1 dpp, with BGE-UDL being the most affected by the temperature. Despite calibrating the instrument, an unexpected decrease in G′ with an increase in angular frequency was observed. However, this unusual behavior was reversed for all formulations after 30 dpp. On the other hand, the G′ and G″ values were lower than those at 20 °C.

At 30 dpp and 20 °C, the values of G′ and G″ for BGE-UDL were still higher than CC-UDL and BC-UDL, as observed for vehicles and formulations at 1 dpp. The values of G′ and G″ at 20 °C for the BGE-UDL formulation increased after 30 dpp. G′ continued to be greater than those G″ for all formulations, indicating that the formulations maintained their structure throughout the test, with the elastic solid character prevailing over the viscous liquid behavior. On the other hand, G′ and G″ values for BC-UDL and CC-UDL were similar, with slightly higher viscoelastic values observed for CC-UDL.

At 30 dpp, the increase in temperature from 20 °C to 37 °C did not result in significant changes in the viscoelastic behavior of both creams with liposomes (BC-UDL and CC-UDL). The values of G′ and G″ for BC-UDL and CC-UDL at 37 °C showed minimal differences compared to those obtained at 20 °C. On the other hand, for BGE-UDL, the values of G′ and G″ slightly decreased at 37 °C compared to those at 20 °C. Despite the increase in temperature, BGE-UDL maintained its elastic solid character throughout all the angular frequency sweep tests, with G′ values remaining higher than G″ values. However, the effect of temperature was less pronounced than that observed for the vehicle without UDL.

**Figure 5:**
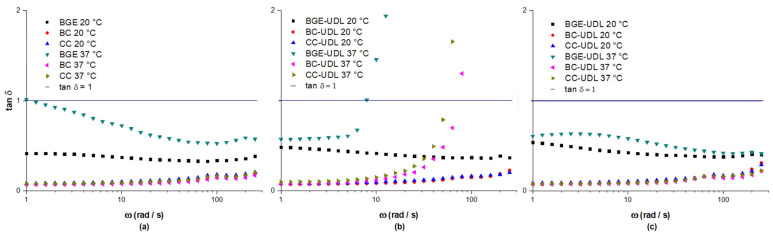
Tan δ for vehicles (**a**) and UDL-loaded formulations at 20 °C and 37 °C for 1 dpp (**b**) and 30 dpp (**c**).

Figure 5 shows that the vehicles and UDL-loaded formulations exhibited an elastic solid character in their viscoelastic behavior, as indicated by values below tan δ = 1. Tan δ represents the relationship between the viscous modulus (G″) and the elastic modulus (G′), and values below 1 indicate that the material behaves more like a solid than a liquid.

The increase in Tan δ observed in Figure 5b for the formulations at 37 °C and 1 dpp, as mentioned earlier in Figure 4d, corresponds to an unexpected behavior that was reversed after 30 dpp. Despite calibrating the instrument to avoid problems in determining the inertia of the measurement unit, the formulations experienced some destabilization at 37 °C, leading to unusual viscoelastic behavior. It is possible that after some time, the dispersed phase stabilizes, and the viscoelasticity can be measured correctly.

## 4. Discussion

In this study, we focused on analyzing the rheological and viscoelastic behavior of topical vehicles used for skin application under varying temperature conditions. Additionally, we investigated the impact of incorporating liposomes into these formulations. Liposomes are known for their potential as drug delivery systems, capable of encapsulating and transporting both lipophilic and hydrophilic drugs. We chose UDL as our drug delivery model because they have the capability to encapsulate and transport various types of drugs through the skin. This makes UDL an ideal choice for delivering cosmeceuticals and active pharmaceutical ingredients (APIs) in topical formulations. While UDL are typically obtained as an aqueous suspension, this presentation is not practical for direct skin application. To make them suitable for topical use, these liposomes need to be incorporated into appropriate vehicles such as creams and gels. Creams and gels are commonly used as topical formulations due to their favorable properties for skin application. Creams are semi-solid emulsions that provide a good balance between water and oil content, making them suitable for both hydrophilic and lipophilic drugs. Gels, on the other hand, have a gel-like consistency, providing enhanced stability and controlled release of active ingredients. By incorporating UDL into creams or gels, we can create formulations that are more convenient and effective for skin application. The vehicles not only help in delivering the liposomes to the skin but also provide other benefits, such as enhanced spreadability, improved skin adherence, and prolonged release of encapsulated drugs or active compounds [25,26,27]. The combination of UDL with creams or gels offers a promising approach for delivering a wide range of drugs and cosmeceuticals through the skin, opening up new possibilities for topical treatments and skin care products.

In a previous study, we analyzed the integrity and mean size of UDL incorporated in the same topical vehicles used in the current study over a period of time [10]. Additionally, we investigated the skin penetration capabilities of these UDL-loaded formulations. The results from this earlier work provided valuable insights into the behavior of UDL in topical vehicles, as well as their ability to penetrate the skin and deliver drugs effectively. This information is essential in understanding the overall performance and potential applications of the UDL-loaded formulations in our current research. We monitored the mean size of UDL using dynamic light scattering (DLS) at different time points: immediately after obtaining UDL, upon their incorporation into the topical vehicles, and after a 7-day period. Notably, no significant differences were observed in the mean size of UDL when they were incorporated into BGE, CC, and TW. This suggested that these vehicles effectively maintained the stability and size of UDL over the study period. However, the incorporation of UDL into BC led to a noteworthy outcome. A significant increase in size was observed, accompanied by the appearance of a second population, which constituted approximately 4% of the total intensity measured by DLS. These findings strongly indicated the occurrence of aggregation in the BC-UDL formulation. After 7 days, the mean size of the main UDL population remained relatively consistent, while the percentage of aggregation increased further. Moreover, we also measured the Z-potential of UDL in the various samples. When UDL were incorporated into CC and TW, the Z-potential module exhibited a slight decrease. Conversely, in BGE-UDL and BC-UDL formulations, the Z-potential was halved after incorporation. These results indicated that the choice of vehicle for UDL incorporation can significantly influence the particle size and stability of UDL over time, with some vehicles showing a greater tendency to promote aggregation. Furthermore, the incorporation of UDL labeled with a fluorescent label facilitated the tracking of their penetration into the skin following their incorporation into the various vehicles. Notably, when UDL were within CC, BGE, and TW formulations, their penetration into the skin exhibited enhancement. In contrast, the incorporation of UDL into BC formulations failed to yield any substantial disparities when compared to the penetration characteristics of free UDL. This disparity in penetration behavior points toward an intricate interplay between the formulation matrix, UDL properties, and the physicochemical attributes of the vehicles, which collectively govern the extent of skin penetration and drug delivery efficiency. The discernible differences in penetration outcomes across various formulations further emphasize the pivotal role of formulation composition in influencing the behavior of UDL within the skin microenvironment.

Rheological behavior plays a crucial role in ensuring the proper flow of materials, especially in the context of topical products. In the field of pharmaceutical technology, rheological behavior holds significant importance for the efficacy of formulations and their ability to provide prolonged action [28]. The rheological behavior of particles in a formulation plays a significant role in determining the mixing and flow characteristics, as well as the physical stability and consumer acceptability of a topical product [22]. Furthermore, certain particle properties can influence the viscosity of the final formulation. Particles that are not solid, such as lipid vesicles, possess internal spaces that can accommodate water, thereby impacting the overall viscosity of the formulation via local alterations. Additionally, the shape of the particles is known to influence their flow properties in the solid state. Therefore, the presence of particles in mixed phases can be expected to affect the rheology of the entire formulation. In topical drug delivery systems, modifying the rheological properties seems to have an impact on the controlled release of drugs from the carriers and affects the bioadhesive properties of the skin [29]. Our current research aimed to understand how the presence of liposomes affects the rheological properties of the topical vehicles, such as their flow behavior and resistance to deformation. We performed comprehensive tests, including frequency sweeps, stress sweeps, and amplitude sweeps, to study the viscoelastic behavior of the formulations. By comparing the results at different temperatures, we could assess the temperature dependence of the viscoelastic properties.

During rheological studies, a shear stress is applied to a particle suspension in a liquid phase, and the response to this stress mainly depends on the particle shape. If the particles slide smoothly over each other, and the response remains unchanged even with increasing shear rate, this behavior is known as Newtonian. However, when the particles have irregular or non-solid shapes and the shear rate is increased to 2000 rpm, it can be anticipated that the particles will not slide smoothly over one another, leading to a non-Newtonian behavior, such as pseudoplastic behavior. This behavior is complex and influenced by factors such as the surface properties of the particles and the surface area. In all cases, the viscosity is expected to exhibit similar changes as the shear rate. Similarly, other factors such as particle size, physical stability [30], concentration of the dispersed phase [31], and the medium viscosity, which is also temperature dependent [11], can influence the rheological behavior. A non-Newtonian fluid is defined as a fluid in which the apparent viscosity varies with the shear rate, either decreasing (shear-thinning) or increasing (shear-thickening). Numerous topical formulations fall under this category, including many carbomer-based gels, pastes, creams, and ointments [32]. The flow behavior observed for BGE (gel), BC (cream), and CC (cream) in the flow analyses was non-Newtonian, and it exhibited pseudoplastic characteristics. Pseudoplastic behavior means that the viscosity of the formulation decreases as the shear rate increases. In other words, these formulations show a decrease in resistance to flow as the applied shear stress or shear rate increases. To quantitatively describe the non-Newtonian flow behavior of these formulations, the Herschel–Bulkley model was chosen to fit the data. The Herschel–Bulkley model is commonly used for non-Newtonian fluids and provides a mathematical description of the flow behavior in terms of consistency index (K), fluidity index (*n*), and yield stress (τ_0_). By fitting the experimental data to the Herschel–Bulkley model, it becomes possible to understand and predict the flow properties of these formulations under different conditions. This information is crucial for the development and optimization of topical products, as it helps in selecting the right formulation and ensuring proper delivery and performance of active ingredients on the skin. Even though TW exhibited Newtonian behavior, which means its viscosity remained constant regardless of the shear rate, the Herschel–Bulkley model still provided an appropriate fit to the data. In the case of TW, since it behaves as a Newtonian fluid, the parameters of the Herschel–Bulkley model would not have significant practical meaning for TW itself. However, using the same model for all the formulations simplifies the analysis and allows for a consistent comparison of the flow behavior among the different vehicles and UDL-loaded formulations. The incorporation of UDL significantly decreased the K for BGE, BC, and CC at both temperatures, indicating that UDL-loaded formulations had lower resistance to flow compared to the vehicles. This decrease in K was more pronounced after 30 dpp in most cases. However, for BC-UDL, the K value was higher at 37 °C after 30 dpp compared to the value obtained for BC-UDL at the same temperature after 1 dpp. Additionally, the R^2^ value for BC-UDL was 0.381 at 37 °C and 0.478 at 20 °C, suggesting that the Herschel–Bulkley model did not fit the data well for BC-UDL formulation after 30 dpp. The data presented in Figure 1e,f further illustrate these observations, showing distortions and deviations of the experimental points. Furthermore, BC-UDL at 30 dpp exhibited noticeable changes in its appearance, characterized by phase separation and a color alteration, signifying a structural deterioration. Creams are emulsions comprising water and lipophilic components such as fats and oils [33]. These lipophilic elements are susceptible to a form of degradation called rancidity [34,35]. Consequently, it is plausible that the instability observed in BC-UDL at 30 dpp was induced by rancidity. Given its instability, it was excluded from the discussion involving the other results obtained at 30 dpp. The BC-UDL deviation from the fit of the model was also supported by previous observations reported by Izquierdo et al. (2020) [10]. The overall results suggest that the incorporation of UDL into BC formulation may destabilize not only the vehicle but also the liposomes, leading to their aggregation. This destabilization and aggregation could be further exacerbated by the aging of the formulation, which may result in changes in the rheological behavior of the formulation. The observed increase in the mean size of UDL within BC-UDL may be related to a decrease in their penetration capability. Larger particles may have reduced permeability through the skin, potentially affecting the efficacy of the formulation. In contrast, for TW-UDL, there were practically no significant changes in the K value when compared to TW. The lack of significant changes in the K value indicates that the incorporation of UDL did not significantly affect the flow behavior of the TW vehicle. The similarity between TW-UDL and the UDL suspension obtained after liposome synthesis also suggests that this formulation may provide a stable environment for UDL. TW was included in this study because it is used as a cosmeceutical vehicle, and our previous work showed that TW-UDL increased the skin penetration of a hydrophilic drug while not affecting UDL size [10].

The viscosity values plotted against the shear rate for gel and creams in this work distinctly indicate a shear-thinning system, which stands as a favorable attribute for effective topical vehicles and formulations [21,22]. Such behavior observed in creams and gels notably enhances their spreadability across the skin surface [36]. Notably, previous researchers have also documented analogous viscosity profiles, aligning with a pseudoplastic flow behavior in formulations incorporating UDL. This finding suggests the feasibility of employing this approach for the development of user-friendly topical formulations [37]. Shear-thinning behavior is characterized by a continuous decrease in viscosity, indicating the progressive release of polymer entanglement under increasing shear stress [21,38,39]. In the case of UDL-loaded formulations, shear-thinning behavior may also result from the disruption of interparticle contacts between particles of the dispersed phase, which likely form a gel. These systems are likely viscoplastic, exhibiting yield stress. However, further studies at lower shear rates are needed to confirm this [40].

In semi-solid systems, characterizing rheology poses a challenge due to their dual manifestation of solid-like and liquid-like attributes within the same material [23,41,42]. To delve into the viscous and elastic behavior of our systems, along with the network structure arising from particle–particle interactions, we conducted oscillation sweep tests [23]. The amplitude sweep test comprises two key steps. Initially, we identify the LVR of the samples, accomplished via a stress sweep at a constant frequency. The LVR represents a stress range in which G* (complex modulus) and the structure of the material remain undisturbed. For our study, the stress sweep was carried out with a stress value (τ) of 1 Pa, selected based on prior research [9], enabling us to pinpoint the linear viscoelastic range. El Kechai et al. (2015) similarly explored the viscoelastic aggregation characteristics of liposomes on hyaluronic acid hydrogels at 37 °C, using a stress value of 10 Pa, corresponding to the linear viscoelastic domain where structural alterations were not irreversible [24]. In our investigation, all tested formulations exhibited distinct LVR within the lower stress threshold (τ < 5 Pa). On the other hand, the frequency sweep test emerges as a valuable instrument for comprehending the interplay between elastic and viscous behaviors in materials, bestowing essential insights into the viscoelastic attributes and structural features of the system. The investigations conducted by [9,43,44] collectively offer valuable insights into the microstructure of Carbopol gels and their corresponding rheological behavior. These studies revealed the presence of a highly cross-linked dense central region in Carbopol gel structures, with hanging free-end parts that engage intensely with other free ends within the surrounding dense core. The irregular and fibrous three-dimensional network detected in low-level polymer content further underscores the intricate nature of the gel microstructure. Notably, the outcomes of this current study align harmoniously with the conclusions from these prior studies, indicating that the viscoelastic behavior of the gel primarily takes on an elastic quality, preserving its internal structure even under the stress of high shear during the frequency sweep test. Stability and resistance to deformation stand as pivotal attributes for topical products, ensuring both consistency and reliability. Additionally, the study by Kamal et al. (2020) on base creams laden with Acyclovir aggregates reinforces the notion that creams lacking aggregates exhibit viscoelastic behavior akin to the results obtained in this study [36]. The surge in G′ during the frequency scan signifies the prevalence of a highly organized emulsified microstructure within the base cream, dominated by cohesive forces between its aqueous and lipid phases. These cohesive forces contribute to the elastic demeanor observed in the creams. Moreover, the results obtained in the current study highlight the temperature-dependent alterations in the viscoelastic traits of the formulations tested, marked by reduced G′ and G″ values and an earlier entry into the fluidity zone at elevated temperatures. Understanding these temperature-induced effects bears paramount importance in the development and refinement of topical products, as they wield the capacity to shape their performance and stability across varying conditions. Finally, the interaction between the elastic and viscous moduli is elucidated via the loss tangent, a parameter that provides insight into the overall viscoelastic nature of a system. Prior investigations by Bucci et al. (2018) revealed that the incorporation of anthocyanin-loaded lipid vesicles (anthocyanin-UDL) into a Carbopol 940 gel did not notably impact the elastic properties of the gel across the explored frequency range [9]. The elastic modulus is intricately linked to the connectivity of the polymeric network, reflecting the count of entities capable of enduring stress, particularly the elastically active network chains. Carvalho et al. (2013) delved into hydrogels formulated with polycarbophil (PP), carbomer homopolymer type A (C971), and type B (C974) at varying concentrations [29]. Their investigation unveiled distinct behavior in the results of frequency sweep tests. Specifically, C971 and PP 0.1% hydrogels, along with C974 and PP 0.1% hydrogels, exhibited a propensity towards viscosity over elasticity, evidenced by the respective moduli of 0.8 Pa vs. 0.2 Pa and 6.0 Pa vs. 3.0 Pa. Notably, both moduli exhibited frequency-dependent characteristics, with G″ surpassing G′ across the selected frequency spectrum. With increasing concentrations, these materials manifested a more solid-like character. The findings of this study underscored that C974 and PP established highly cross-linked hydrogels, while C971 gels exhibited a more fluidic nature, attributed to the lightly cross-linked network crafted by this polymer.

Collectively, the findings of this study and the correlated research underscore the significance of grasping the microstructure and rheological behavior of topical formulations, as they wield a pivotal influence on their effectiveness and suitability for diverse applications within cosmeceutical and pharmaceutical products. The choice of vehicle can significantly impact the stability, rheological behavior, and drug penetration capability of the final product. The findings highlight the importance of carefully selecting the appropriate vehicle and formulation conditions when incorporating UDL or other liposomal drug delivery systems.

## 5. Conclusions

This study provides valuable insights into the rheological and viscoelastic characteristics of topical formulations with potential applications in both the cosmeceutical and pharmaceutical industries. The incorporation of UDL into various gel and cream-based vehicles designed for topical use did not alter their non-Newtonian behavior. UDL-loaded formulations exhibited pseudoplastic behavior immediately after the dispersion of liposomes and continued to do so even after a month of their incorporation, even under temperature variations ranging from 20 to 37 °C. However, it is important to note that the incorporation of UDL led to a significant reduction in the resistance to flow compared to the vehicle alone at both studied temperatures. This effect was more pronounced after 30 days post-production in most cases. Furthermore, all formulations maintained their elastic solid behavior over a viscous liquid state upon the incorporation of UDL at both 20 and 37 °C. Nonetheless, it is worth highlighting that these formulations require a stabilization period for the liposomal phase after dispersion within the vehicle matrix. Additionally, our study identified a need for optimization of the BC-UDL formulation to prevent deterioration after 30 days post-production. Understanding these effects can be valuable for researchers and formulators in optimizing the performance of cosmeceutical and pharmaceutical products containing liposomes, thus enhancing their potential therapeutic benefits. Further investigations and studies are essential to fine-tune the formulation and achieve the desired stability and drug delivery characteristics.

## Figures and Tables

**Table 1:** Composition of topical vehicles listed according to the International Nomenclature of Cosmetic Ingredients (INCI).

Vehicle	Composition (INCI)
Base gel with essence (BGE)	Water, propylene glycol, acrylates/C10-30 alkyl acrylate crosspolymer, triethanolamine, methylparaben, imidazolidinyl urea, and fragrance.
Base cream (BC)	Water, cyclopentasiloxane and dimethicone crosspolymer, palmitoyl glycine, mineral oil, cetearyl alcohol and ceteareth-20, polyacrylamide, C13-14 isoparaffin, laureth-7, carbomer, triethanolamine, lanolin, cetyl alcohol, methylparaben, propylparaben, dimethicone, tocopheryl acetate, disodium EDTA, methylchloroisothiazolinone, and methylisothiazolinone
Commercial formulation cream-based (CC)	Water, cyclopentasiloxane and dimethicone crosspolymer, cyclopentasiloxane, urea, propylene glycol, hyaluronic acid, alcohol, sodium PCA, arginine, lepidium meyenii root extract, zingiber officinale root extract, palmitoyl glycine, mineral oil, cetearyl alcohol and ceteareth-20, polyacrylamide, C13-14 isoparaffin, laureth-7, carbomer, triethanolamine, lanolin, cetyl alcohol, fragrance, methylparaben, propylparaben, dimethicone, tocopheryl acetate, disodium EDTA, allantoin, retinyl palmitate, hexyl cinnamal, alpha-isomethyl ionone, methylchloroisothiazolinone, and methylisothiazolinone
Thermal water (TW)	Aqua, thermal spring water, propylene glycol, ginkgo biloba leaf extract, chamomilla recutita flower extract, hamamelis virginiana leaf extract, tilia platyphyllos flower extract, phenoxyethanol, ethylhexylglycerin, boric acid, triethanolamine, may contain D-limonene, linalool, benzyl salicylate, and citronellol

**Table 2:** Herschel–Bulkley model parameters for the empty vehicles.

Sample	Temp. (°C)	τ_0_ (Pa)	K (Pa × s^n^) ^a^	*n* ^a^	R^2^
BGE	20	13	21.17 ± 1.11	0.351 ± 0.002	0.867
	37	28	32.65 ± 1.97	0.243 ± 0.013	0.945
BC	20	107	121.05 ± 1.11	0.242 ± 0.006	0.980
	37	110	135.37 ± 1.08	0.210± 0.006	0.979
CC	20	92	111.55 ± 1.24	0.247 ± 0.007	0.980
	37	89	109.98 ± 1.17	0.230 ± 0.007	0.972
TW	20	0.0019	0.0031 ± 0.0007	0.906 ± 0.045	0.931
	37	0.00018	0.00021 ± 0.00030	0.992 ± 0.024	0.982

^a^ Mean ± SD (*n* = 3).

**Table 3:** Herschel–Bulkley model parameters for the UDL-loaded vehicles at 1 dpp.

Sample	Temp. (°C)	τ_0_ (Pa)	K (Pa × s^n^) ^a^	*n* ^a^	R^2^	*p* ^b^
BGE-UDL	20	17	17.31 ± 1.27	0.289 ± 0.012	0.952	<0.01
	37	22	23.93 ± 1.06	0.185 ± 0.013	0.871	<0.0005
BC-UDL	20	50	53.85 ± 1.04	0.238 ± 0.007	0.972	<0.0001
	37	51	54.45 ± 1.17	0.226 ± 0.008	0.968	<0.0001
CC-UDL	20	34	38.11 ± 1.78	0.252 ± 0.007	0.977	<0.0001
	37	45	48.17 ± 1.12	0.212 ± 0.005	0.986	<0.0001
TW-UDL	20	0.0011	0.0013 ± 0.0009	0.963 ± 0.021	0.986	ns
	37	0.00028	0.00033 ± 0.00007	1.040 ± 0.051	0.934	ns

^a^ Mean ± SD (*n* = 3). ^b^ UDL-loaded formulations were compared to the vehicle without UDL using one-way ANOVA and Dunnett’s multiple comparisons test.

**Table 4:** Herschel–Bulkley model parameters for the UDL-loaded vehicles at 30 dpp.

Sample	Temp. (°C)	τ_0_ (Pa)	K (Pa × s^n^) ^a^	*n* ^a^	R^2^	*p* ^b^
BGE-UDL	20	10	10.85 ± 1.24	0.347 ± 0.012	0.895	<0.0001
	37	11	11.89 ± 1.14	0.333 ± 0.007	0.987	<0.0001
BC-UDL	20	43	43.95 ± 1.51	0.108 ± 0.042	0.478	<0.0001
	37	70	71.15 ± 1.33	0.226 ± 0.008	0.381	<0.0001
CC-UDL	20	42	43.45 ± 1.29	0.256 ± 0.008	0.974	<0.0001
	37	41	42.16 ± 1.87	0.239 ± 0.007	0.972	<0.0001
TW-UDL	20	0.0050	0.0054 ± 0.0009	0.891 ± 0.042	0.923	<0.05
	37	0.00040	0.00018 ± 0.00007	1.004 ± 0.067	0.885	ns

^a^ Mean ± SD (*n* = 3). ^b^ UDL-loaded formulations were compared to the vehicle without UDL using one-way ANOVA and Dunnett’s multiple comparisons test.

## Data Availability

The data presented in this study are available in this article.
